# Facing Thyroid Nodules in Paediatric Patients Previously Treated with Radiotherapy for Non-Thyroidal Cancers: Are Adult Ultrasound Risk Stratification Systems Reliable?

**DOI:** 10.3390/cancers13184692

**Published:** 2021-09-18

**Authors:** Arnoldo Piccardo, Francesco Fiz, Gianluca Bottoni, Camilla De Luca, Michela Massollo, Ugo Catrambone, Luca Foppiani, Monica Muraca, Alberto Garaventa, Pierpaolo Trimboli

**Affiliations:** 1 Department of Nuclear Medicine, E.O. “Ospedali Galliera”, 16128 Genoa, Italy; arnoldo.piccardo@galliera.it (A.P.); gianluca.bottoni@galliera.it (G.B.); michela.massollo@galliera.it (M.M.); 2 Department of Health Sciences (DISSAL), Università di Genova, 16132 Genoa, Italy; camilla.deluca93@gmail.com; 3 Department of Surgery, E.O. “Ospedali Galliera”, 16128 Genoa, Italy; ugo.catrambone@galliera.it; 4 Department of Internal Medicine, E.O. “Ospedali Galliera”, 16128 Genoa, Italy; luca.foppiani@galliera.it; 5 Epidemiology and Biostatistic Unit and DOPO Clinic, IRCCS Istituto Giannina Gaslini, 16147 Genoa, Italy; monicamuraca@gaslini.org; 6 Department of Oncology, IRCCS Istituto Giannina Gaslini, 16147 Genoa, Italy; albertogaraventa@gaslini.org; 7 Clinic for Endocrinology and Diabetology, Regional Hospital of Lugano, Ente Ospedaliero Cantonale, 6900 Lugano, Switzerland; Pierpaolo.Trimboli@eoc.ch; 8 Faculty of Biomedical Sciences, Università della Svizzera Italiana (USI), 6900 Lugano, Switzerland

**Keywords:** thyroid nodules, paediatrics, radiotherapy, risk assessment, ultrasonography, DTC

## Abstract

**Simple Summary:**

The risk of thyroid nodules harbouring cancer has been evaluated, in adults, using specific ultrasound criteria. However, it is unclear whether such evaluation can be translated in paediatric patients. In this study, we tested the effectiveness of three known risk evaluation systems in children with thyroid nodules and with a history of radiation exposure. We found that these systems are reliable in confirming or ruling out cancer in most cases, except when evaluating very small nodules (<1 cm). For these reasons, these risk criteria should be adopted to account for the reduced size of malignant lesions when evaluating paediatric subjects.

**Abstract:**

Thyroid nodule ultrasound-based risk stratification systems (US-RSSs) have been successfully used in adults to predict the likelihood of malignancies. However, their applicability to the paediatric population is unclear, especially in children with a history of radiation exposure, who are at a higher cancer risk. We tested the efficacy of three US-RSSs in this setting by retrospectively applying three classification systems (ACR-TIRADS, ATA and EU-TIRADS) to all paediatric patients referred for thyroid nodules and with a radiation exposure history. We compared the results with a reference standard (pathology or 36-month follow-up); sensitivity, specificity, positive and negative predictive values (PPV and NPV) and accuracy were calculated. A total of 52 patients were included; fourteen of them (27%) had papillary thyroid cancer (PTC) at the final histology. No significant differences across the US-RSSs were detected; specificity (range 95–97%) and NPV (range 88–93%) were particularly elevated. However, ACR-TIRADS, ATA and EU-TIRADS did not indicate the need for a biopsy in six (42.8%), seven (50%) and eight (57%) cases of PTC; in five cases, this lack of indication was due to a small (<1 cm) nodule size. In conclusion, US-RSSs show a high NPV and specificity in paediatric patients, whereas the cytology indication could be improved by reconsidering the dimensional criterion.

## 1. Introduction

Thyroid nodules are fairly uncommon among paediatric subjects [1]. However, whenever a thyroid lesion is identified in children and teenagers, it does bear a higher likelihood of malignancy, which can be as high as 20–25%, when compared with the adult counterpart (5%) [2,3]. Some risk factors may increase the probability of developing thyroid nodules in children, including iodine deficiency, prior radiation exposure and several genetic syndromes. 

In particular, childhood cancer survivors who were treated for their non-thyroidal primary malignancy with radiation therapy (RT) represent a population at risk. This group includes survivors of Hodgkin lymphoma, leukaemia, neuroblastoma and central nervous system tumours [2,4,5]. In fact, the history of malignancy and the radiation exposure can represent synergic factors for the development of a second malignant neoplasm, particularly differentiated thyroid cancer (DTC) [6,7,8]. 

Neck ultrasonography is the first-line imaging procedure, which is able to identify and classify the risk of the thyroid nodules [9,10,11]. Adult-based neck US risk stratification systems (RSSs) have been developed in recent years to integrate the US features in an effort to improve diagnostic accuracy and as an aid in the stratification of the risk of malignancy [9,12,13]. 

However, few studies are available about the reliability of these systems in paediatric age, and conflicting results have been reported about the accuracy of the adult US-RSSs. 

In particular, little can be said about the efficacy of US-RSSs when it comes to stratifying the risk of malignancy in patients with a history of previous radiotherapy for oncological reasons who need a strict follow-up after the identification of thyroid nodules [7,14]. This could bear particular relevance considering that an early identification of DTC could avoid a more advanced presentation that, in paediatric patients, can imply extrathyroidal extension and metastases. 

The most recent guidelines seem to be concordant in considering those patients at high risk of developing DTCs with thyroid nodules and a previous history of irradiation regardless of the neck ultrasonography features and dimensions. Indeed, while fine-needle aspiration cytology (FNA) can be generally recommended in adults with nodules sized at least 1 cm, in the paediatric population, this procedure is indicated even for small nodules [9,15]. Current US-RSSs do not include young age and previous history of RT as risk factors [16]. Indeed, whether the RSS reliability is concordant with the one that is generally reported remains to be clarified [17].

The aim of our study was to: (1) evaluate the diagnostic performance of the principal neck US classification systems (ACR-TIRADS, ATA and EU-TIRADS) in a selected paediatric population of patients previously treated with radiotherapy, (2) test the malignancy prevalence of each category delineated by US-RSSs and (3) evaluate whether these neck US systems are able to correctly select nodules for FNA.

## 2. Materials and Methods

We retrospectively analysed all paediatric patients consecutively referred to our centre (Galliera Hospital) for FNA of a thyroid nodule between 1 January 2012 and 31 December 2017. Before FNA, all patients underwent thyroid US and were tested for TSH, free-T4, free-T3 and calcitonin. Additionally, thyroid scintigraphy (TS) was performed only in the case of suppressed TSH levels [2]. Patients were excluded only if US data had not been retrieved in the local picture archiving and communication system (PACS). Then, only patients with thyroid nodules and previously treated with radiotherapy (RT) for primary paediatric non-thyroidal tumours were included in our study. The institutional review board (Comitato Etico Regionale Liguria, Registration Number: 326/2020-DB id 10315) approved this retrospective study.

### 2.1. Neck Ultrasonography

Thyroid US was performed using a LOGIQ S8 (General Electric Medical Systems) with a 9 to 15 MHz linear probe. All imaging procedures were performed in combination with a clinical visit by 3 expert physicians (A.P., G.B., M.M.). For all patients, a greyscale and colour Doppler imaging data were acquired.

### 2.2. Ultrasound Risk Stratification Systems

All thyroid nodules were retrospectively risk stratified according to the principal US-RSSs (i.e., ACR-TIRADS, ATA and EU-TIRADS). Indications for FNA were ascertained depending on risk classes identified by each US-RSS. More in general, FNA could be indicated depending on US features and nodule dimensions.

### 2.3. Imaging Review and Interpretation

Neck US images we retrieved from the PACS and then visually analysed by 2 reviewers (AP, PT) unaware of patients’ data and final outcome. The inter- and intra-reader variabilities in identifying the classes of each US-RSS were previously tested in a different set of 30 paediatric patients with thyroid nodules and showed excellent agreement (Cohen’s κ, 0.82 [95% CI, 0.68–0.91]). In case of interpretation disagreement, the final diagnosis was achieved after a consensus meeting with a third expert (GB).

For statistical purposes, each thyroid nodule with US features corresponding to the last class of each US-RSS (i.e., EU-TIRADS 5, ACR-TIRAD5 and ATA High-risk) was regarded as positive. Prevalence of malignancy was calculated as the percent of nodules in each class that were confirmed as DTC at the final histology.

### 2.4. Reference Standard 

Cytology according to the Italian consensus of cytopathology was adopted as the gold standard. The first edition, used until 2014, considered 5 classes, with a single indeterminate category, while the second edition included 6 classes, of which 2 were indeterminate [18,19]. However, for all these patients, a US follow-up of at least 36 months was available. In case of surgery, histopathology of the resected nodule represented the standard of trough.

### 2.5. Statistical Analysis 

Sensitivity, specificity, positive and negative predictive values (PPV and NPV) and accuracy were calculated for each system. Differences in categorical variables between groups were analysed using the chi-square test or Fisher’s exact test as appropriate.

The prevalence of malignancy was calculated as the ratio between the number of DTCs in each class and the total number of DTCs.

The proportion of FNA that would not have been indicated by the various systems in patients with a diagnosis of DTC was compared using the pairwise chi-square test.

## 3. Results

**Figure 1:**
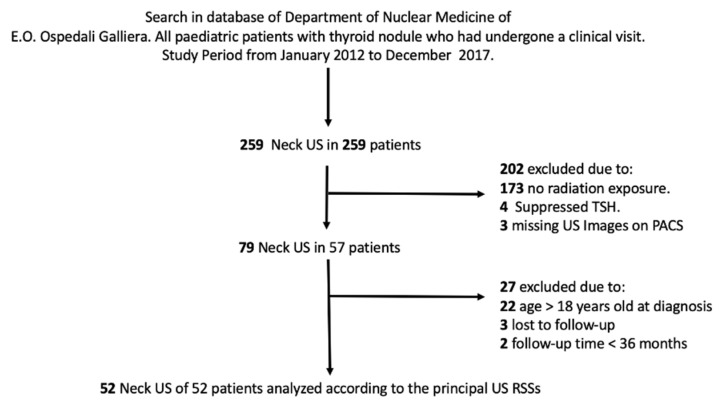
Flow chart illustrating the selection of the patients.

During the study period, we evaluated 259 paediatric patients with thyroid nodules who had undergone neck US at our department. Out of these patients, 52 were selected for the present study according to our inclusion criteria (Figure 1), and their principal characteristics are summarised in Table 1.

Among these 52 patients, 19 underwent surgery because of a symptomatic nodular goitre (n = 3), indeterminate cytology (n = 4) and cytology suspicious (n = 3) or consistent with DTC (n = 9). Finally, 14 papillary cancers (27%) were histologically confirmed (Table 1).

The diagnostic performances of each US-RSS in terms of sensitivity, specificity, positive and negative predictive values (PPV and NPV) and accuracy in identifying DTCs are summarised in Table 2. No significant differences across these systems were observed.

When benign nodules were evaluated according to the three US-RSSs, the principal classes in which they were included were EU-TIRADS 3 by EU-TIRADS, TR3 by ACR-TIRADS and “Low suspicion” by ATA.

When PTCs were considered, the most represented classes were EU-TIRADS 5 by EU-TIRADS (71.4%), TR5 by ACR-TIRADS (71.4%) and “High suspicion” by ATA (64.2%).

**Figure 2:**
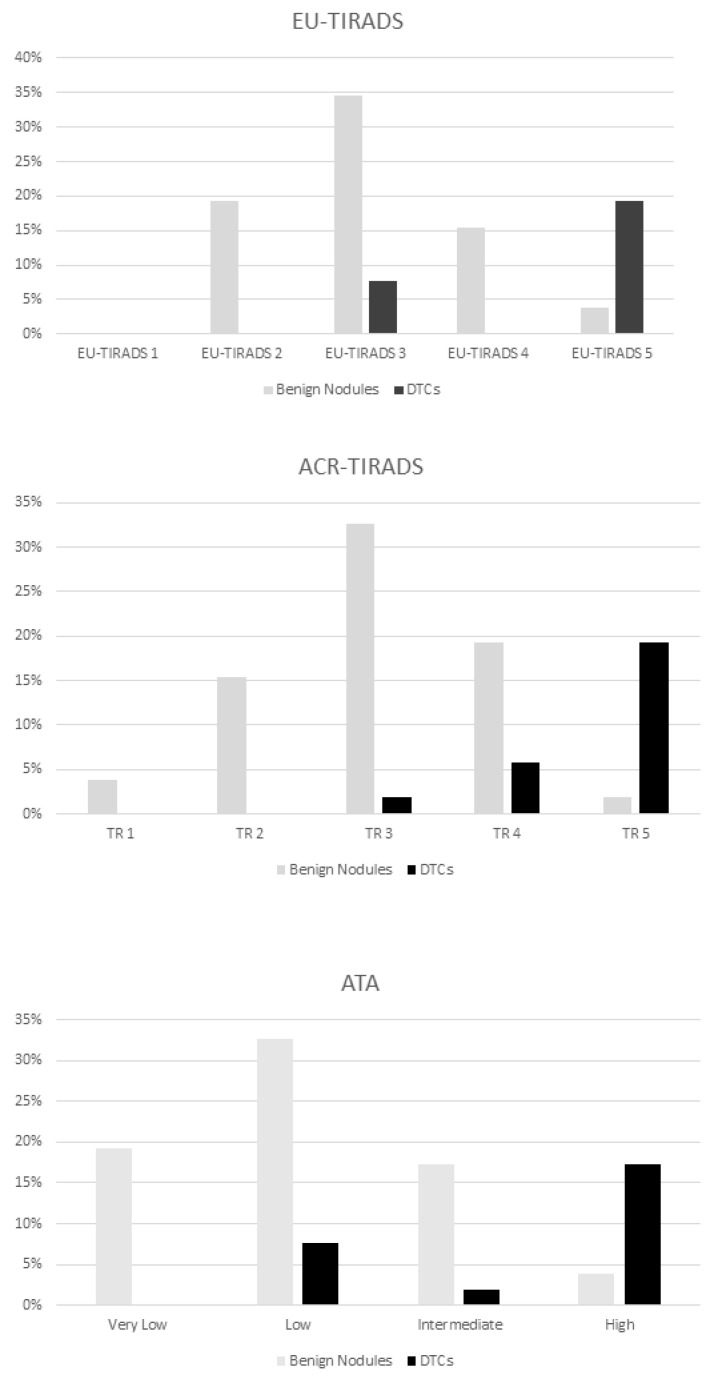
Distribution of benign and malignant thyroid nodules among the categories of the three US-RSSs.

A statistical comparison showed no significant differences in the benign lesion and PTC distributions among the three systems (Figure 2).

At evaluation of the risk of malignancy of each category of the various US-RSSs, we found that EU-TIRADS 5, TR5 and ATA High suspicion presented a DTC percentage of 71, 71 and 64, respectively (Table 3). Conversely, no DTCs were found in the lowest categories.

**Figure 3:**
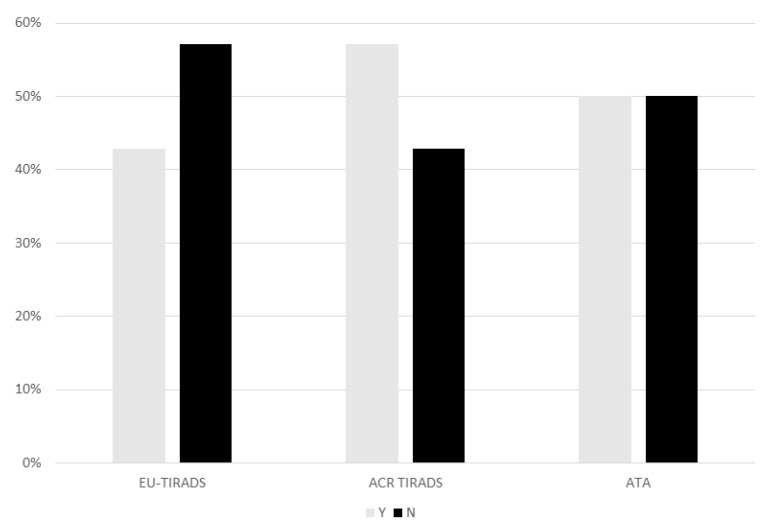
Indication for fine-needle aspiration (FNA) cytology in papillary thyroid cancers according to the criteria set by the 3 US-RSSs.

When FNA indication in patients subsequently diagnosed with papillary thyroid cancer on histopathology was analysed according to the US-RSSs, EU-TIRADS missed eight cases (57%), ACR TIRADS six (42.8%) cases and ATA seven cases (50%) (Figure 3). The lack of indication for FNA was principally related (five patients) to the small dimensions (<1 cm) of the malignant thyroid nodules.

## 4. Discussion

The pivotal role of neck ultrasonography in the identification and evaluation of the malignant potential of thyroid nodules, as well as in FNA guiding, has been recognised in the most recent paediatric guidelines [20,21]. Strikingly however, no specific US features to tell apart benign from malignant nodules have been identified, and no dedicated scoring system has been proposed [2]. Some papers have investigated the role of US-RSSs in the paediatric population with conflicting results [22,23]. To our knowledge, this is the first study testing US-RSSs in a selected population of paediatric patients with a history of neck radiation exposure.

In our population, a little less than one third of the radiotherapy-treated patients had developed a DTC; this result is well in line with the data reported in the existing literature [24,25].

Moreover, in this particular setting of patients in which FNA is “a priori” indicated due to the high prevalence of malignancy, we showed that the different US-RSSs can rule out DTCs, having a high NPV ranging from 89 (ATA) up to 91% (ACR TIRADS). Indeed, this finding, similar to that reported in a recent paper comparing the two American systems (i.e., ATA and ACR TIRADS) [23], may have a particular impact on the ability to monitor these subjects at increased risk with reliable, non-invasive procedures. In particular, by contributing to sparing futile and repeated invasive procedures, it can help in reducing stress and anxiety for patients who have been previously heavily pre-treated for non-thyroidal cancer.

We found that, by considering the highest category of each system as positive and the remaining ones as negative, the specificity is very high (from 95 to 97%), with a very low number of false positive US findings. Our data, in this regard, are concordant with a recent meta-analysis by Kim et al. showing that, by using the same interpretation, the pooled specificity of ACR TIRADS in paediatric patients is 97% [26]. On the contrary, the prevalence of DTC within the thyroid nodules classified in the highest categories is even higher than that reported by Kim and colleagues, ranging from 64 for ATA to 71% for ACR TIRADS, without significant differences among the US-RSSs. Generally, we found that all US stratification systems are reliable methods to identify DTCs, and that their diagnostic performances are adequate and higher than those reported by the meta-analysis by Kim et al. [26]. This discrepancy could be related to the higher prevalence of malignancy in the irradiated population and to the histopathology which is exclusively papillary thyroid cancer (PTC). Indeed, a large part of the studies considered in this meta-analysis [22,23,27] excluded patients with a history of radiation exposure and included patients with thyroid cancer other than PTC (10%) for which the US-RSSs are often not reliable enough [17,28].

When we analysed the ability of the three US-RSSs in identifying which thyroid nodule should be investigated by means of FNA, we found that, by rigorously applying the dimensional criteria, all three systems did not provide a proper indication for FNA in more than 40% of DTC patients (from 43 to 57%). Indeed, no significant differences were observed among the three systems. This finding supports the yet unproved indication for FNA in these particular patients with micronodules (i.e., <1 cm) reported in the most recent guidelines [2]. Indeed, there is some evidence that childhood cancer survivors tend to have, on average, smaller thyroid tumours [29]. In addition, it must be underlined that three out of five patients with DTCs smaller than 1 cm already showed loco-regional lymph node involvement (i.e., two with N1a, and one with N1b). This aggressive biological behaviour of small DTCs, which is expected in paediatric patients, should be carefully considered in the drafting of dedicated US paediatric risk stratification systems.

Some limitations should be underlined. First, the retrospective nature of this study may be associated with selection biases that could have affected our results. However, the DTC prevalence and the time from irradiation to DTC onset are in agreement with those estimates by the ATA guidelines [2]. Second, the sample size and the number of DTCs were limited; however, this is the first study evaluating the role of US-RSSs in paediatric patients with a well-known history of irradiation exposure, and overall, the number of patients included in this study is in line with others evaluating non-irradiated patients [23,27,30]. Finally, only for 19 out of 52 patients was a histopathological confirmation available. However, for all patients, cytological results and at least 3 years of clinical and US follow-up were available.

## 5. Conclusions

We found that the American and European US-RSSs have a high NPV and specificity in detecting DTCs, having the possibility to rule out malignancy even in this particular subgroup of high-risk patients. In addition, the DTC prevalence among the highest system categories was very high, achieving 71%. However, according to all three of the aforementioned US-RSSs, the majority of PTCs would not be selected for FNA. This result is related to the size cut-offs proposed by US-RSSs for indicating FNA rather than US features. Both users of thyroid US-RSSs and panellists of the next TIRADSs should be aware of the present findings.

## Figures and Tables

**Table 1:** Characteristics of the patients.

Variable	Subjects Included (n = 52)
Sex	
Female, n. (%)	32 (61.5)
Male, n. (%)	20 (38.5)
Age on nodule diagnosis, median (IQR), years	17 (15–18)
<15 years, n. (%)	11 (21.1)
≥15 years, n. (%)	41 (78.9)
Age on irradiation, median (IQR), year	5 (3–7)
<5 years, n. (%)	24 (46.1)
≥5 years, n. (%)	28 (53.9)
Time from RT to thyroid nodule diagnosis median (IQR), year	11 (8–14)
<10 years, n. (%)	16 (30.8)
≥10 years, n. (%)	36 (69.2)
Nodule dimensions, median (IQR), mm	13 (11–22)
<10 mm, n. (%)	7 (13.4)
10–15 mm, n. (%)	26 (50.0)
16–20 mm, n. (%)	5 (9.6)
>20 mm, n. (%)	16 (30.7)
Thyroid cytology *	
Tir 2, n. (%)	36 (69.2)
Tir 3, n. (%)	2 (3.8)
Tir 3b, n.(%)	2 (3.8)
Tir 4, n. (%)	3 (5.7)
Tir 5, n. (%)	9 (17.3)
Thyroidectomy	
Yes, n. (%)	19 (36.5)
No, n. (%)	33 (63.5)
Pathology	
Papillary thyroid carcinoma, n. (%)	14 (73.)
Follicular thyroid carcinoma, n. (%)	0 (0)
Follicular hyperplasia	4 (21.0)
Follicular adenoma	1 (5.3)
Age on DTC diagnosis, median (IQR), years	15 (14–18)
<15 years, no. (%)	4 (28.5)
≥15 years, no. (%)	10 (71.5)
Time from RT to DTC diagnosis, median (IQR), year	11 (10–12)
<10 years, no. (%)	3 (21.4)
≥10 years, no. (%)	10 (78.6)
Clinico-pathological classification **	
T1, no. (%)	12 (85.7)
T2, no. (%)	2 (14.3)
N0, no. (%)	5 (35.7)
N1a, no. (%)	7 (50.0)
N1b, no. (%)	2 (14.3)
M0, no. (%)	14 (100)

Legend: * According to the Italian Consensus Working Group [reference]. ** This feature included all histopathological findings and pre-surgical imaging. IQR: Interquartile range, RT: radiation treatment, DTC: differentiated thyroid carcinoma.

**Table 2:** Diagnostic performances of the US-RSSs.

US-RSSs	ACR-TIRADS	EU-TIRADS	ATA	*p*-VALUE (ACR vs. EU TIRADS)	*p*-VALUE (ACR TIRADS vs. ATA)	*p*-VALUE (EU TIRADS vs. ATA)
Sensitivity	71%	71%	64%	0.66	0.4	0.68
Specificity	97%	95%	95%	0.56	0.56	1
NPV	91%	90%	88%	0.69	0.47	0.75
PPV	91%	83%	82%	0.53	0.47	0.92
Accuracy	91%	88%	87%	0.87	0.83	0.72

Legend US-RSSs: Ultra-sound risk stratification systems, ACR: American college of radiology. TI-RADS: Thyroid imaging reporting and data system, EU: European thyroid association, ATA: American thyroid association, NPV: negative predictive value, PPV: positive predictive value.

**Table 3:** Frequency of malignancy according to the various US-RSSs.

Category	Prevalence of Malignancy
EU-TIRADS	
EU-TIRADS 2	0%
EU-TIRADS 3	4/14 (29%)
EU-TIRADS 4	0%
EU-TIRADS 4	10/14 (71%)
ACR TIRADS	
TR 1	0%
TR 2	0%
TR 3	1/14 (7%)
TR 4	3/14 (22%)
TR 5	10/14 (71%)
ATA	
Benign	0%
Very low	0%
Low	4/14 (29%)
Intermediate	1/14 (7%)
High	9/14 (64%)

## Data Availability

Data regarding the present study are available at the authors’ institution and can be obtained upon reasonable request.

## References

[1] Cimbek E.A., Polat R., Sonmez B., Beyhun N.E., Dinc H., Saruhan H., Karaguzel G. (2021). Clinical, sonographical, and pathological findings of pediatric thyroid nodules. Eur. J. Pediatr..

[2] Francis G.L., Waguespack S.G., Bauer A.J., Angelos P., Benvenga S., Cerutti J.M., Dinauer C.A., Hamilton J., Hay I.D., Luster M. (2015). Management Guidelines for Children with Thyroid Nodules and Differentiated Thyroid Cancer. Thyroid.

[3] Richman D.M., Cherella C.E., Smith J.R., Modi B.P., Zendejas B., Frates M.C., Wassner A.J. (2021). Clinical utility of sonographic features in indeterminate pediatric thyroid nodules. Eur. J. Endocrinol..

[4] Elisei R., Romei C., Vorontsova T., Cosci B., Veremeychik V., Kuchinskaya E., Basolo F., Demidchik E.P., Miccoli P., Pinchera A. (2001). RET/PTC rearrangements in thyroid nodules: Studies in irradiated and not irradiated, malignant and benign thyroid lesions in children and adults. J. Clin. Endocrinol. Metab..

[5] Shulan J.M., Vydro L., Schneider A.B., Mihailescu D.V. (2018). Role of biomarkers in predicting the occurrence of thyroid neoplasms in radiation-exposed children. Endocr. Relat. Cancer.

[6] Jarzab B., Handkiewicz-Junak D. (2007). Differentiated thyroid cancer in children and adults: Same or distinct disease?. Hormones.

[7] Piccardo A., Foppiani L., Puntoni M., Hanau G., Calafiore L., Garaventa A., Arlandini A., Villavecchia G., Bianchi P., Cabria M. (2012). Role of low-cost thyroid follow-up in children treated with radiotherapy for primary tumors at high risk of developing a second thyroid tumor. Q. J. Nucl. Med. Mol. Imaging.

[8] Iglesias M.L., Schmidt A., Ghuzlan A.A., Lacroix L., Vathaire F., Chevillard S., Schlumberger M. (2017). Radiation exposure and thyroid cancer: A review. Arch. Endocrinol. Metab..

[9] Haugen B.R., Alexander E.K., Bible K.C., Doherty G.M., Mandel S.J., Nikiforov Y.E., Pacini F., Randolph G.W., Sawka A.M., Schlumberger M. (2016). 2015 American Thyroid Association Management Guidelines for Adult Patients with Thyroid Nodules and Differentiated Thyroid Cancer: The American Thyroid Association Guidelines Task Force on Thyroid Nodules and Differentiated Thyroid Cancer. Thyroid.

[10] Mistry R., Hillyar C., Nibber A., Sooriyamoorthy T., Kumar N. (2020). Ultrasound Classification of Thyroid Nodules: A Systematic Review. Cureus.

[11] Grant E.G., Tessler F.N., Hoang J.K., Langer J.E., Beland M.D., Berland L.L., Cronan J.J., Desser T.S., Frates M.C., Hamper U.M. (2015). Thyroid Ultrasound Reporting Lexicon: White Paper of the ACR Thyroid Imaging, Reporting and Data System (TIRADS) Committee. J. Am. Coll. Radiol..

[12] Russ G., Bonnema S.J., Erdogan M.F., Durante C., Ngu R., Leenhardt L. (2017). European Thyroid Association Guidelines for Ultrasound Malignancy Risk Stratification of Thyroid Nodules in Adults: The EU-TIRADS. Eur. Thyroid J..

[13] Tessler F.N., Middleton W.D., Grant E.G., Hoang J.K., Berland L.L., Teefey S.A., Cronan J.J., Beland M.D., Desser T.S., Frates M.C. (2017). ACR Thyroid Imaging, Reporting and Data System (TI-RADS): White Paper of the ACR TI-RADS Committee. J. Am. Coll. Radiol..

[14] Miao S., Jing M., Sheng R., Cui D., Lu S., Zhang X., Jing S., Zhang X., Shan T., Shan H. (2020). The analysis of differential diagnosis of benign and malignant thyroid nodules based on ultrasound reports. Gland. Surg..

[15] Sakorafas G.H., Mastoraki A., Lappas C., Safioleas M. (2010). Small (<10 mm) thyroid nodules; how aggressively should they be managed?. Onkologie.

[16] Russ G., Trimboli P., Buffet C. (2021). The New Era of TIRADSs to Stratify the Risk of Malignancy of Thyroid Nodules: Strengths, Weaknesses and Pitfalls. Cancers.

[17] Castellana M., Castellana C., Treglia G., Giorgino F., Giovanella L., Russ G., Trimboli P. (2020). Performance of Five Ultrasound Risk Stratification Systems in Selecting Thyroid Nodules for FNA. J. Clin. Endocrinol. Metab..

[18] Fadda G., Basolo F., Bondi A., Bussolati G., Crescenzi A., Nappi O., Nardi F., Papotti M., Taddei G., Palombini L. (2010). Cytological classification of thyroid nodules. Proposal of the SIAPEC-IAP Italian Consensus Working Group. Pathologica.

[19] Nardi F., Basolo F., Crescenzi A., Fadda G., Frasoldati A., Orlandi F., Palombini L., Papini E., Zini M., Pontecorvi A. (2014). Italian consensus for the classification and reporting of thyroid cytology. J. Endocrinol. Investig..

[20] Izquierdo R., Shankar R., Kort K., Khurana K. (2009). Ultrasound-guided fine-needle aspiration in the management of thyroid nodules in children and adolescents. Thyroid.

[21] Buryk M.A., Simons J.P., Picarsic J., Monaco S.E., Ozolek J.A., Joyce J., Gurtunca N., Nikiforov Y.E., Feldman Witchel S. (2015). Can malignant thyroid nodules be distinguished from benign thyroid nodules in children and adolescents by clinical characteristics? A review of 89 pediatric patients with thyroid nodules. Thyroid.

[22] Creo A., Alahdab F., Al Nofal A., Thomas K., Kolbe A., Pittock S.T. (2018). Ultrasonography and the American Thyroid Association Ultrasound-Based Risk Stratification Tool: Utility in Pediatric and Adolescent Thyroid Nodules. Horm. Res. Paediatr..

[23] Martinez-Rios C., Daneman A., Bajno L., van der Kaay D.C.M., Moineddin R., Wasserman J.D. (2018). Utility of adult-based ultrasound malignancy risk stratifications in pediatric thyroid nodules. Pediatr. Radiol..

[24] Gupta A., Ly S., Castroneves L.A., Frates M.C., Benson C.B., Feldman H.A., Wassner A.J., Smith J.R., Marqusee E., Alexander E.K. (2013). A standardized assessment of thyroid nodules in children confirms higher cancer prevalence than in adults. J. Clin. Endocrinol. Metab..

[25] Niedziela M. (2006). Pathogenesis, diagnosis and management of thyroid nodules in children. Endocr. Relat. Cancer.

[26] Kim P.H., Yoon H.M., Hwang J., Lee J.S., Jung A.Y., Cho Y.A., Baek J.H. (2021). Diagnostic performance of adult-based ATA and ACR-TIRADS ultrasound risk stratification systems in pediatric thyroid nodules: A systematic review and meta-analysis. Eur. Radiol..

[27] Polat Y.D., Ozturk V.S., Ersoz N., Anik A., Karaman C.Z. (2019). Is Thyroid Imaging Reporting and Data System Useful as an Adult Ultrasonographic Malignancy Risk Stratification Method in Pediatric Thyroid Nodules?. J. Med. Ultrasound.

[28] Trimboli P., Castellana M., Piccardo A., Romanelli F., Grani G., Giovanella L., Durante C. (2021). The ultrasound risk stratification systems for thyroid nodule have been evaluated against papillary carcinoma. A meta-analysis. Rev. Endocr. Metab. Disord..

[29] Clement S.C., Lebbink C.A., Klein Hesselink M.S., Teepen J.C., Links T.P., Ronckers C.M., van Santen H.M. (2020). Presentation and outcome of subsequent thyroid cancer among childhood cancer survivors compared to sporadic thyroid cancer: A matched national study. Eur. J. Endocrinol..

[30] Uner C., Aydin S., Ucan B. (2020). Thyroid Image Reporting and Data System Categorization: Effectiveness in Pediatric Thyroid Nodule Assessment. Ultrasound Q..

